# Comparative study of long-term efficacy of spinal fusion surgery and non-surgical treatment for chronic radicular lumbar disease

**DOI:** 10.1590/1806-9282.20240902

**Published:** 2025-03-17

**Authors:** Yuanmei Li, Huijuan Song, Hongzhen Zhou, Jungui Zhou, Zhou Zhou

**Affiliations:** 1 Southern Medical University, Nanfang Hospital, Department of Rehabilitation Medicine – Guangzhou, China.; 2 Southern Medical University, Nanfang Hospital, Department of Nursing – Guangzhou, China.

**Keywords:** Spinal fusion, Lumbar vertebrae, Surgery, Follow-up studies, Rehabilitation

## Abstract

**OBJECTIVE:**

The objective of this study was to compare the long-term efficacy of spinal fusion surgery versus non-surgical treatment for chronic radicular lumbar spondylopathy.

**METHODS:**

A total of 93 patients with chronic radicular lumbar spondylopathy admitted to our hospital from February 2020 to February 2021 were randomly divided into a non-surgical group (n=46, conservative treatment) and a surgical group (n=47, spinal fusion surgery). Efficacy, recurrence rate, pain index, lumbar function recovery, and quality of life were evaluated and compared between the groups.

**RESULTS:**

The surgical group had a higher total effective rate (97.87 vs. 86.96%, p<0.05) and a lower recurrence rate after 1 year (4.26 vs. 21.74%, p<0.05) compared to the non-surgical group. There was no significant difference in visual analog scale scores for lower back pain and lower limb pain between the groups before treatment and for the first 3 days (p>0.05). However, at 1 month, 3 months, and 1 year after treatment, the visual analog scale scores were significantly lower in the surgical group (p<0.05). The Japanese Orthopedic Association score showed no significant difference before treatment (p>0.05) but increased significantly in the surgical group after 1 month, 3 months, and 1 year (p<0.05). Similarly, there was no significant difference in the Short Form 36-Item Health Survey score before treatment (p>0.05), but the surgical group had significantly higher scores after 1 year (p<0.05).

**CONCLUSION:**

Spinal fusion surgery offers better long-term efficacy than non-surgical treatment for chronic radicular lumbar spondylopathy. It effectively alleviates lower back and limb pain, promotes lumbar function recovery, and improves quality of life, making it a recommended treatment option.

## INTRODUCTION

Chronic nerve root lumbar disease is a common orthopedic disease caused by compression and stimulation of the lumbar nerve root, resulting in root symptoms such as pain, numbness, and sensory abnormalities^1^. Thickened fibrous rings and yellow ligaments, as well as protruding discs which cause soft tissue compression, lead to a decrease in the volume of the spinal canal, compressing the spinal cord and nerve roots, causing ischemia and hypoxia in the spinal cord and nerve roots. The main clinical diagnoses include lumbar disc herniation, narrowing of the nerve root canal, and lateral recess of the lumbar canal. The symptoms of nerve root lumbar disease include pain on both sides of the lumbar spinous process, numbness in the lower limbs, and dysfunction of urination, which affect the patient's labor ability and quality of life.

Due to the varying severity of the clinical manifestations of chronic nerve root lumbar disease, diverse treatment methods have been developed. Currently, patients mainly have surgical and non-surgical treatment methods. Generally, patients with a shorter course of disease first consider non-surgical treatment methods. Common non-surgical treatment methods include conservative treatments such as massage, traction, physiotherapy, and non-steroidal anti-inflammatory drugs, which have the characteristics of minimal trauma and low risk and can alleviate and cure the symptoms in most patients. Surgical treatment is chosen when conservative treatment is ineffective^2^. Spinal fusion is a neurosurgical or orthopedic surgical technique that connects two or more vertebrae together, which can quickly alleviate the clinical symptoms of patients^3,4^. Currently, most studies on the surgical treatment and non-surgical treatment of chronic nerve root lumbar disease are focused on the evaluation of short-term efficacy, while there are few follow-up studies on long-term efficacy.

Based on this, in order to investigate the optimal treatment plan for patients, this study selected 93 patients with chronic nerve root lumbar disease admitted to our hospital from February 2020 to February 2021 and compared the efficacy of different treatment methods to provide reference for clinical practice.

## METHODS

### General information

This study has been approved by the Ethics Committee of Nanfang Hospital of Southern Medical University, and informed consent has been obtained from the patients or their family members. A total of 93 patients with chronic radicular lumbar spine disease admitted to our hospital from February 2020 to February 2021 were randomly assigned to a surgical group (47 patients receiving spinal fusion surgery) and a non-surgical group (46 patients receiving conservative treatment) using a random number table method. There were no significant differences in the general data between the two groups, p>0.05, as shown in Table 1.

**Table 1:** Comparison of general information between the two groups.

Group	Sex (n, %)		Average age (years)	Average duration of illness (years)	Occupation		
	Male	Female			Farmer	Worker	Athlete
Surgery group (n=47)	24	23	58.41±5.84	2.54±0.46	18	21	8
Non-surgery group (n=46)	22	24	58.94±5.89	2.69±0.53	20	19	7
χ^2^/t	0.097		0.436	1.459	0.261		
P	0.755		0.664	0.148	0.878		

Inclusion criteria were as follows: (1) all patients who were diagnosed with chronic radicular lumbar disease^1^; (2) patients and their families who had a thorough understanding of the research content and voluntarily signed the informed consent form; (3) patients who had not undergone lumbar surgery; and (4) patients’ age>18 years.

Exclusion criteria were as follows: (1) patients with lumbar instability or slippage; (2) coagulopathy; (3) mental disorders; and (4) severe heart and lung disease.

### Intervention method

The non-surgical group received conservative treatment, including: (1) If there were no contraindications, using ibuprofen and other anti-inflammatory analgesics to control inflammation and pain, administering intravenous mannitol and other liquid therapeutic drugs to reduce edema and relieve nerve root compression, and suggesting bed rest and keeping the waist warm; (2) placing the patient in a supine position for manual massage of the lumbar spine and sciatic nerve, followed by applying rhythmic pressure on the waist; (3) using a protective gear to fix the chest and pelvis in a supine position, adjusting traction force to 10-25 kg based on the protrusion position, and applying lumbar spine traction for about 30 min daily for 30 days; and (4) performing functional exercises on the lumbar and back muscles and allowing the patient to get out of bed with waist protection after acute symptoms subside.

The surgical group underwent lumbar fusion via an intervertebral foramen endoscope. The procedure included: (1) routine disinfection and administration of general or epidural anesthesia in a prone position; (2) inserting a guide wire into the lumbar spinous process and placing a puncture needle and an intervertebral foramen endoscope; (3) cutting the yellow ligament to expose the nerve root, separating it gently to reveal the diseased intervertebral disc tissue, and removing the pressure-causing nucleus pulposus tissue; (4) cutting the intervertebral disc in the foramen area and implanting a polyetheretherketone intervertebral fusion device; (5) connecting a titanium rod and screw fixation device to the vertebrae, using bone substitutes or growth factors to promote vertebral fusion; and (6) placing a drainage tube and suturing the incision, removing the drainage tube 24-48 h post-surgery, and administering antibiotics.

### Observational indicators

Clinical efficacy was evaluated by categorizing outcomes as cure (complete symptom disappearance and lumbar mobility recovery), effective (improved symptoms and lumbar mobility), or ineffective (no improvement or worsening). The overall effective rate was calculated as the sum of cure and effective rates. Recurrence rates were compared over 2 years via outpatient visits and calls. Pain severity was measured using the visual analog scale (VAS) (0-10 scale) before treatment and at 1 month, 3 months, and 1 year post-treatment. The lumbar function was assessed with the Japanese Orthopedic Association (JOA) score (0–29 scale) at the same intervals. The quality of life was evaluated using the Short Form 36-Item Health Survey (SF-36) survey before and after treatment, covering eight dimensions and totaling 100 points^5–7^.

### Statistical analysis

SPSS 23.0 software was used to analyze and process data. Count data were presented as percentages and tested using the chi-square test. Measurement data were expressed as ÷±s and were tested using the t-test, with p<0.05 considered statistically significant.

## RESULTS

### Comparison of the clinical efficacy of the study subjects

The results showed that the total effective rate of the surgery group (46/47: 97.87%) was higher than that of the non-surgery group (40/46: 86.96%), p<0.05. Additionally, the recurrence rate after 1 year in the surgery group (2/47: 4.26%) was lower than that in the non-surgery group (10/46: 21.74%), p<0.05.

### Comparison of pain scores in the study subjects

The results showed that there was no significant difference in lower back pain VAS scores and lower limb pain VAS scores between the study subjects before treatment for the first 3 days (p>0.05). However, 1 month after treatment, the VAS scores of the study subjects decreased, and the non-surgery group had lower VAS scores than the surgery group (p<0.05). At 3 months and 1 year after treatment, the surgery group had significantly lower VAS scores than the non-surgery group (p<0.05) (Table 2).

**Table 2:** Comparison of pain scores and lumbar function scores in the study subjects (points, $\overset{-}{\chi }$ ± s).

		Surgery group (n=47)	Non-surgery group (n=46)	t	p
Lower back pain VAS scores	A	5.71±0.84	5.67±0.76	0.241	0.810
	B	4.86±0.81	4.38±0.72	3.018	0.003
	C	3.24±0.75	3.94±1.03	3.752	<0.001
	D	1.44±0.68	1.89±0.74	3.055	0.003
Lower limb pain VAS scores	A	6.48±1.57	6.54±1.62	0.181	0.856
	B	4.82±1.06	4.27±1.01	2.561	0.012
	C	2.46±0.82	3.08±0.93	3.412	0.001
	D	1.63±0.58	1.95±0.66	2.485	0.015
Lumbar function scores	A	14.38±3.49	14.15±3.42	0.321	0.749
	B	17.92±3.19*	16.15±3.25*	2.651	0.009
	C	18.87±3.56*	17.26±3.61*	2.165	0.033
	D	22.93±3.17*	21.33±3.21*	2.418	0.018

A: 3 days before treatment; B: 1 month after treatment; C: 3 months after treatment; D: 1 year after treatment; VAS: visual analog scale.

* Compared with A (3 days before treatment), P<0.05.

### Comparison of lumbar function scores between the two groups

The results showed that there was no significant difference in JOA scores between the study subjects before treatment (p>0.05). However, after 1 month, 3 months, and 1 year of treatment, the scores increased, and the surgical group had significantly higher scores than the non-surgical group (p<0.05) (Table 2).

### Comparison of the quality of life among the study subjects

Table 3 shows that there was no significant difference in SF-36 scores between the study subjects before treatment (p>0.05). However, 1 year after treatment, both groups demonstrated improvements, with the surgical group experiencing significantly higher scores than the non-surgical group (p<0.05).

**Table 3:** Comparison of quality of life scores in the study subjects (points, $\overset{-}{\chi }$ ± s).

		Surgery group (n=47)	Non-surgery group (n=46)	t	p
Physical health	Before treatment	64.17±6.41	63.84±6.38	0.249	0.804
	One year after treatment	73.84±7.38*	69.12±6.91*	3.182	0.002
Physical pain	Before treatment	66.24±6.62	65.79±6.57	0.329	0.743
	One year after treatment	73.75±7.37*	70.13±7.01*	2.426	0.017
Physical role function	Before treatment	67.84±6.78	68.35±6.83	0.361	0.719
	One year after treatment	75.42±7.54*	71.36±7.13*	2.667	0.009
Vitality	Before treatment	68.14±6.81	67.83±6.78	0.220	0.826
	One year after treatment	74.24±7.42*	70.46±7.04*	2.519	0.014
Overall health	Before treatment	65.86±6.58	66.31±6.63	0.329	0.743
	One year after treatment	73.21±7.32*	68.75±6.87*	3.028	0.003
Social function	Before treatment	69.58±6.95	68.86±6.88	0.502	0.617
	One year after treatment	80.13±8.01*	72.36±7.23*	4.907	<0.001
Mental health	Before treatment	61.18±6.11	58.97±5.89	1.775	0.079
	One year after treatment	68.52±6.85*	61.89±6.18*	4.897	<0.001
Emotional role function	Before treatment	64.58±6.45	65.22±6.52	0.476	0.635
	One year after treatment	78.21±7.82*	73.56±7.35*	2.953	0.004

* Compared with ‘Before treatment’, p<0.05.

## DISCUSSION

Chronic nerve root lumbar disease is caused by the rupture of the fibrous ring of the lumbar intervertebral disc and cauda equina nerve and protrusion of the nucleus pulposus, which stimulate or compress the nerve root. It is characterized by slow onset, long duration, and severe symptoms^8,9^, with long-term desk work, prolonged standing, and external impacts being the important causes. Symptoms include lower back and leg pain, intermittent limping, and radiating pain in the lower limbs, often accompanied by numbness, osteoporosis, and hypertension, significantly impacting the quality of life and work ability^10^.

Non-surgical treatments, such as bed rest, traction, massage, administering anti-inflammatory and analgesic drugs, and reducing nerve root edema, are often chosen for initial or mild cases. However, these methods provide only temporary relief and do not address the root cause. With advancements in surgical technology, lumbar fusion surgery under endoscopic guidance is increasingly preferred for its ability to precisely target the lesion, preserve normal lumbar structure, and shorten recovery time.

Spinal fusion surgery, especially minimally invasive endoscopic surgery, has become more common due to its advantages of minimal trauma, less bleeding, and rapid recovery^11^. Wu et al. showed that patients receiving fusion therapy had significant improvements in clinical symptoms and a higher rate of returning to work compared to those receiving conservative therapy^12^. This study found that surgical patients had a higher effective rate and lower recurrence rate over a 2-year follow-up compared to non-surgical patients. Additionally, post-treatment SF-36 scores, which measure the quality of life, were significantly higher in the surgical group, with no pre-treatment differences between the groups.

Non-surgical treatments can reduce disc pressure and alleviate nerve root compression through methods like traction and massage, but they do not repair the diseased tissue. These methods, based on doctors’ experience, often result in long treatment times and high recurrence rates^13–18^. In contrast, surgical intervention aims to remove lumbar nerve root compression by exposing and removing the protruding disc and implanting a bone graft material for spinal stability, resulting in quick and clear treatment effects.

Pain relief is a key criterion for evaluating chronic radicular lumbar disease treatment efficacy. Xin et al. showed that fusion surgery effectively alleviates pain and improves motor function^19,20^. This study also found that surgical patients had a higher effective rate and lower recurrence rate over a 2-year follow-up, with higher post-treatment SF-36 scores compared to non-surgical patients. Non-surgical treatments, while less invasive, are non-targeted and provide only temporary relief, leading to low efficacy and high recurrence rates. In contrast, lumbar fusion surgery under intervertebral foramen endoscopy minimizes trauma and damage to the ligamentum flavum and lamina, removes the nucleus pulposus without harming nerves or muscles, and preserves spinal stability. The small incision reduces iatrogenic damage, preserves bone and ligament integrity, alleviates short-term pain, and promotes functional recovery^21,22^.

This study has the following limitations: First, the sample size may be limited, resulting in the statistical results being unstable which may not fully reflect the overall treatment effect. Second, differences in surgical procedures and postoperative care may affect efficacy evaluation, and further standardization of operating procedures is needed. In addition, factors such as individual patient differences and follow-up time may also introduce deviations. To solve these problems, it is recommended to expand the sample size, unify surgical and nursing standards, and extend follow-up time to improve the accuracy and reliability of the study results. At the same time, consideration can be given to introducing diversity factors during the analysis to more comprehensively evaluate the treatment effect under different conditions.

## CONCLUSION

Compared to non-surgical treatment, lumbar fusion under intervertebral foramen endoscopy effectively alleviates lower back and limb pain, promotes lumbar function recovery, and positively impacts overall health and social function in patients with chronic nerve root lumbar disease. Its long-term efficacy is superior, making it a valuable treatment option.
